# Folie à deux: contagious mental illness? Report of a clinical case

**DOI:** 10.1192/j.eurpsy.2022.2053

**Published:** 2022-09-01

**Authors:** A. Costa, S. Jesus, M. Almeida, J. Alcafache

**Affiliations:** Baixo Vouga Hospital Centre - EPE, Psychiatry And Mental Health Department, Aveiro, Portugal

**Keywords:** Induced delusional disorder, shared psychosis, folie à deux, Shared delusion

## Abstract

**Introduction:**

Folie à deux is a clinical condition that was first described in 19th century. It is a psychotic disorder in which two closely associated individuals share a similar delusional system. However, folie à deux is still a matter of study and debate today as it remains a challenge for psychiatrists.

**Objectives:**

The aim of this article is to report a clinical case of folie à deux, between na inducer son and an induced mother. Review the nosological significance of folie à deux and to explore the disorder among patients with psychosis.

**Methods:**

Search in the PubMed/MedLine and Medscape databases with the following key words: folie à deux; shared psychosis; shared delusion.

**Results:**

We presente a case of folie à deux between na inducer son 28 years old and the induced, his mother. They were found to be sharing similiar delusional beliefs. The patient has assumed the role of “man of the house” since his father’s death.

**Conclusions:**

Many years after it was first described, folie à deux is still an interesting and challenging disorder to psychiatrists. Its recognition and correct referral for a rare diagnosis, such as folie a deux, are extremely important.

**Disclosure:**

No significant relationships.

